# Genetic polymorphisms in glycolytic pathway are associated with the prognosis of patients with early stage non-small cell lung cancer

**DOI:** 10.1038/srep35603

**Published:** 2016-10-21

**Authors:** Shin Yup Lee, Cheng Cheng Jin, Jin Eun Choi, Mi Jeong Hong, Deuk Kju Jung, Sook Kyung Do, Sun Ah Baek, Hyo Jung Kang, Hyo-Gyoung Kang, Sun Ha Choi, Won Kee Lee, Yangki Seok, Eung Bae Lee, Ji Yun Jeong, Kyung Min Shin, Sukki Cho, Seung Soo Yoo, Jaehee Lee, Seung Ick Cha, Chang Ho Kim, You Mie Lee, In-Kyu Lee, Sanghoon Jheon, Jae Yong Park

**Affiliations:** 1 Lung Cancer Center, Kyungpook National University Medical Center, Daegu 41404, Republic of Korea; 2 Departments of Internal Medicine, School of Medicine, Kyungpook National University, Daegu 41944, Republic of Korea; 3 Department of Biochemistry and Cell Biology, School of Medicine, Kyungpook National University, Daegu 41944, Republic of Korea; 4 BK21 Plus KNU Biomedical Convergence Program, Department of Biomedical Science, Kyungpook National University, Daegu 41944, Republic of Korea; 5 Cell and Matrix Research Institute, School of Medicine, Kyungpook National University, Daegu 41944, Republic of Korea; 6 Biostatistics Center, School of Medicine, Kyungpook National University, Daegu 41944, Republic of Korea; 7 Department of Thoracic Surgery, School of Medicine, Kyungpook National University, Daegu 41944, Republic of Korea; 8 Department of Pathology, School of Medicine, Kyungpook National University, Daegu 41944, Republic of Korea; 9 Department of Radiology, School of Medicine, Kyungpook National University, Daegu 41944, Republic of Korea; 10 Department of Thoracic and Cardiovascular Surgery, Seoul National University School of Medicine, Seoul 13620, Republic of Korea; 11 College of Pharmacy, Kyungpook National University, Daegu, 41566, Republic of Korea

## Abstract

This study was conducted to investigate whether polymorphisms of genes involved in glycolysis are associated with the prognosis of patients with non-small cell lung cancer (NSCLC) after surgical resection. Forty-four single nucleotide polymorphisms (SNPs) of 17 genes in glycolytic pathway were investigated in a total of 782 patients with NSCLC who underwent curative surgical resection. The association of the SNPs with overall survival (OS) and disease free survival (DFS) were analyzed. Among the 44 SNPs investigated, four SNPs (*ENO1* rs2274971A > G, *PFKM* rs11168417C > T, *PFKP* rs1132173C > T, *PDK2* rs3785921G > A) were significantly associated with survival outcomes in multivariate analyses. When stratified by tumor histology, three SNPs (*ENO1* rs2274971A > G, *PFKM* rs11168417C > T, and *PDK2* rs3785921G > A) were significantly associated with OS and/or DFS only in squamous cell carcinoma, whereas *PFKP* rs1132173C > T exhibited a significant association with survival outcomes only in adenocarcinoma. When the four SNPs were combined, OS and DFS decreased as the number of bad genotypes increased (*P*trend = 8 × 10^−4^ and 3 × 10^−5^, respectively). Promoter assays showed that *ENO1* rs2274971G allele had significantly higher promoter activity compared to the rs2274971A allele. The four SNPs, especially *ENO1* rs2274971A > G, may be useful for the prediction of prognosis in patients with surgically resected NSCLC.

Lung cancer is the leading cause of cancer deaths worldwide, with an average 5-year survival rate of 16%^1^. Although surgery is the best treatment modality for potentially curing early stage non-small cell lung cancer (NSCLC), a large proportion of patients die from disease recurrence. Pathologic stage is the most important predictor of prognosis after surgical resection of NSCLC. However, patients with the same pathologic stage have different risk of recurrence and death^2^. Therefore, many studies have focused on prognostic biomarkers for more precise prognostication of patients after surgery.

Metabolic reprogramming is one of the hallmarks of cancer^3^. Dependence on glycolysis under normoxic conditions, or aerobic glycolysis which is often referred to as the Warburg effect, is regarded as one of the characteristic changes in tumor cellular bioenergetics^4^. Given that glycolysis is much less efficient than oxidative phosphorylation in the production of adenosine triphosphate, cancer cells adapt to this disadvantage by increasing glucose uptake to facilitate increased glucose consumption^4^. The increased glucose uptake in cancer cells compared to in normal cells is widely exploited in positron emission tomography imaging using ^18^F-fluorodeoxyglucose (^18^F-FDG). The major oncogenes such as *RAS*, *MYC*, and *HIF-1α* are key inducers of glycolysis and glucose transporters, whereas tumor suppressor TP53 is known to suppress glycolytic flux^5^,^6^. In addition to energy production, the metabolic intermediates of glycolysis play a pivotal role in the production of building blocks such as amino acids, lipids, and nucleic acids in proliferating cancer cells, thus conferring a growth advantage^4^,^5^. The accumulation of glycolytic intermediates promotes the pentose phosphate pathway, resulting in the generation of NADPH, a major reducing agent important in redox homeostasis and drug detoxifying reactions in cancer cells^7^. Glycolytic enzymes and metabolic intermediates of aerobic glycolysis also have important functions beyond glycolysis to facilitate the growth and survival of cancer cells^8^. For example, the mitochondrial membrane-bound hexokinase 2 antagonizes the pro-apoptotic machinery, providing a survival advantage to cancer cells^9^. For pyruvate kinase, the M2 isoform has been reported to promote tumorigenesis and provide a selective growth advantage for tumor cells^10^. In addition, a glycolytic intermediate fructose-1, 6 bisphosphate plays an anti-apoptotic role in cancer cells by maintaining the cytochrome c in a reduced, inactive state^11^. Taken together, the oncogenic regulation of aerobic glycolysis and various roles of the components of the glycolytic pathway highlight the biological significance of glycolysis in cancer.

In this study, we hypothesized that polymorphisms of genes involved in glycolysis affect energy production, macromolecular biosynthesis and other non-glycolytic functions in cancer cells, thus playing a role in determining the prognosis of patients with lung cancer. To test this hypothesis, we evaluated the association of genetic variants in the glycolytic pathway with the prognosis of lung cancer patients undergoing surgical resection.

## Results

### Patient characteristics and clinical predictors

The clinical and pathologic characteristics of the patients and association with overall survival (OS) and disease-free survival (DFS) are shown in Table 1. Univariate analysis showed that age (log-rank *P* [*P*_L-R_] = 2 × 10^−3^), sex (*P*_L-R_ = 4 × 10^−4^), smoking status (*P*_L-R_ = 3 × 10^−4^), diabetes (*P*_L-R_ = 0.03), and pathologic stage (*P*_L-R_ = 1 × 10^−11^) were significantly associated with OS. Only pathologic stage was significantly associated with DFS (*P*_L-R_ = 2 × 10^−15^).

### Associations between SNPs and survival outcomes

**Figure 1:**
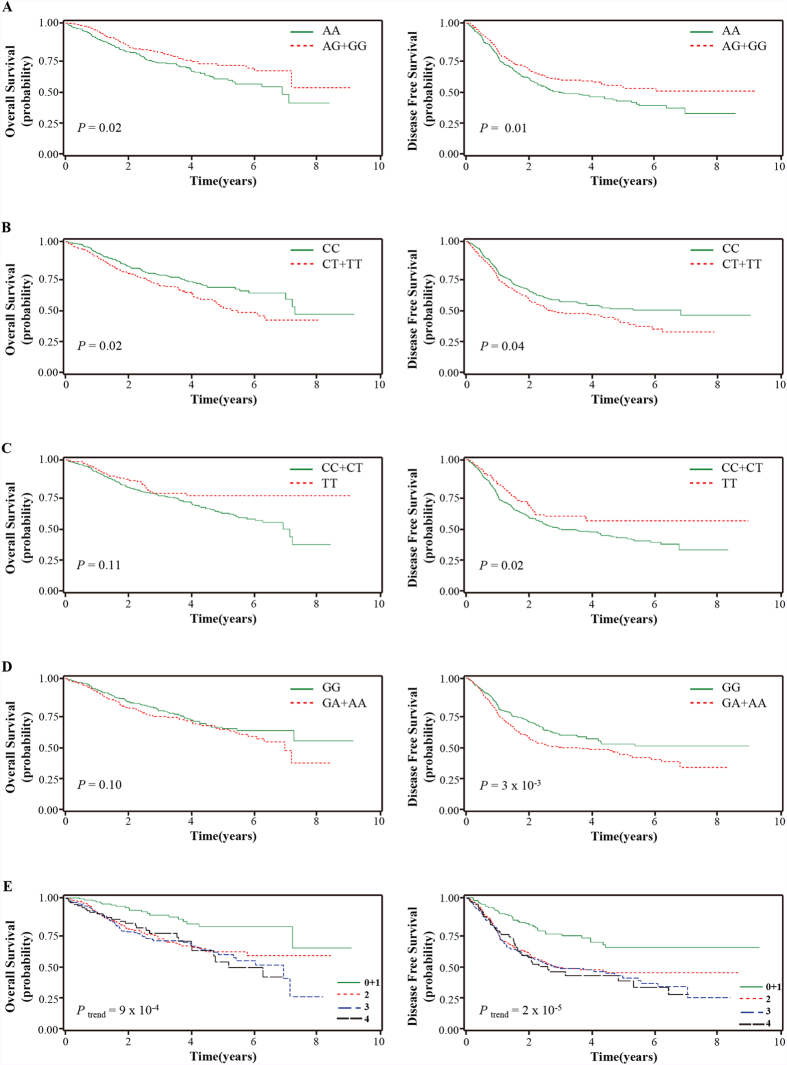
Overall survival and disease free survival according to *ENO1* rs2274971A > G (**A**), *PFKM* rs11168417C > T (**B**), *PFKP* rs1132173C > T (**C**), and *PDK2* rs3785921G > A (**D**) genotypes, and the number of bad genotypes of the four SNPs (**E**). *P* values in the multivariate Cox proportional hazard model.

Among the genotyped 54 single nucleotide polymorphisms (SNPs), 44 were analyzed in this study after excluding one SNP with a <95% call rate and nine showing deviation from Hardy-Weinberg equilibrium. The SNP ID, gene information, base change, minor allele frequencies, and *P* values for OS and DFS of the 44 SNPs are shown in Supplementary Table 1. Among the 44 SNPs analyzed, four SNPs (Enolase 1 [*ENO1*] rs2274971A > G, phosphofructokinase, muscle [*PFKM*] rs11168417C > T, phosphofructokinase, platelet [*PFKP*] rs1132173C > T, and pyruvate dehydrogenase kinase, isoenzyme 2 [*PDK2*] rs3785921G > A) were significantly associated with survival outcomes. *ENO1* rs2274971A > G and *PFKM* rs11168417C > T were significantly associated with OS (adjusted hazard ratio [aHR] = 0.70, 95% confidence interval [CI] = 0.52–0.95, *P* = 0.02; and aHR = 1.44, 95% CI = 1.09–1.91, *P* = 0.01, under the dominant model, respectively) and DFS (aHR = 0.73, 95% CI = 0.58–0.92, *P* = 7 × 10^−3^; and aHR = 1.25, 95% CI = 1.00–1.56, *P* = 0.05, under the dominant model, respectively, Table 2 and Fig. 1A–D). *PFKP* rs1132173C > T and *PDK2* rs3785921G > A were significantly associated with DFS (aHR = 0.73, 95% CI = 0.55–0.98, *P* = 0.03, under the recessive model; and aHR = 1.40, 95% CI = 1.12–1.75, *P* = 3 × 10^−3^, under the dominant model, respectively). We further analyzed the effect of the four SNPs on survival outcomes according to tumor histology. Three SNPs (*ENO1* rs2274971A > G, *PFKM* rs11168417C > T, and *PDK2* rs3785921G > A) were significantly associated with OS and/or DFS in squamous cell carcinoma (SCC) but not in adenocarcinoma (AC), whereas *PFKP* rs1132173C > T exhibited a significant association with survival outcomes in AC but not in SCC (Table 3 and Supplementary Figure 1A–D). The significant difference in survival outcomes according to tumor histology was determined by a test for heterogeneity (*P* for heterogeneity test < 0.05, for OS and/or DFS in all four SNPs).

### Combined effects of the four SNPs on survival outcomes

We performed an exploratory analysis to investigate the combined effects of *ENO1* rs2274971A > G, *PFKM* rs11168417C > T, *PFKP* rs1132173C > T, and *PDK2* rs3785921G > A, which showed a significant association with survival outcomes in the individual SNP analysis. Because *ENO1* rs2274971 AA, *PFKM* rs11168417 CT + TT, *PFKP* rs1132173 CC + CT, and *PDK2* rs3785921 GA + AA were associated with worse survival outcomes, we considered these as bad genotypes and then evaluated their combined effects by grouping the patients based on the number of bad genotypes in each patient. Because there was no death in the zero bad genotype group, we combined the zero and one bad genotype groups. Compared with the reference group that had 0–1 bad genotypes, OS and DFS decreased as the number of bad genotypes increased (*P*trend = 8 × 10^−4^ for OS; and *P*trend = 3 × 10^−5^ for DFS; Table 4 and Fig. 1E). In SCC, the combined analysis of *ENO1* rs2274971 AA, *PFKM* rs11168417 CT + TT, and *PDK2* rs3785921 GA + AA, which were associated with worse survival outcomes in SCC, also showed a significant decrease in OS and DFS as the number of bad genotypes increased (*P*trend = 2 × 10^−5^ for OS; and *P*trend = 5 × 10^−7^ for DFS; Table 4 and Supplementary Figure 1E).

### Effect of rs2274971A > G on promoter activity of *ENO1*

**Figure 2:**
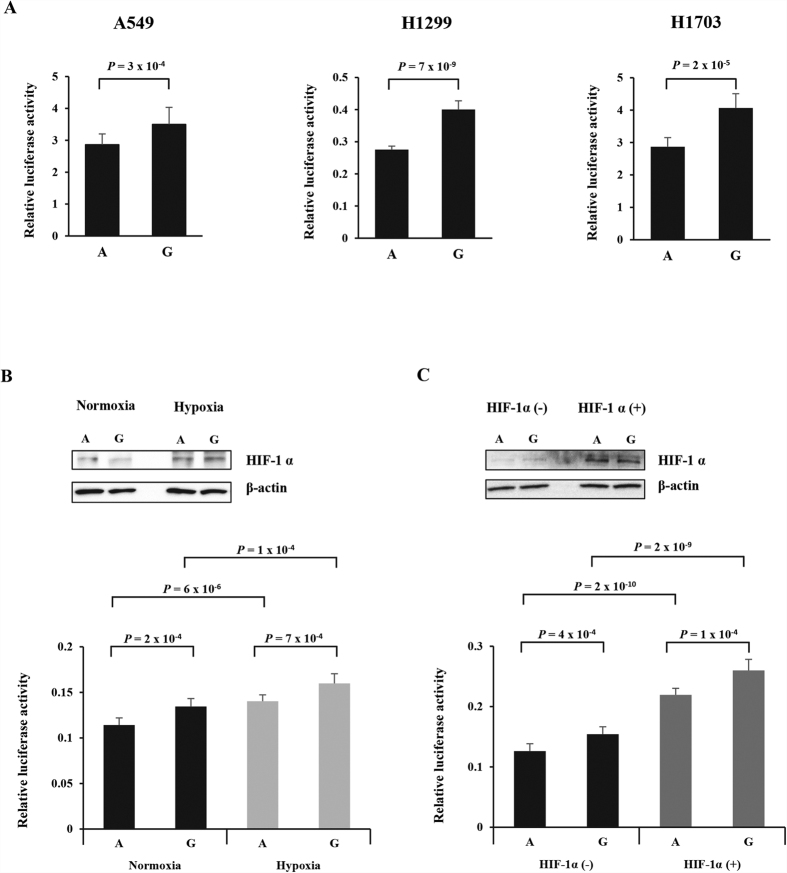
Functional analysis of the *ENO1* rs2274971A > G by dual luciferase reporter assay. NSCLC cells (A549, H1299, and H1703) were transfected with pRL-SV40 and pGL3-basic-*ENO1* rs2274971A or G allele-containing constructs (**A**). At 24 h posttransfection, A549 cells were exposed to hypoxia for 24 h (**B**). For HIF-1α overexpression, we cotransfected pRL-SV40 and pGL3-basic-*ENO1* constructs into A549 cells with pEGFP-HIF-1α (**C**). Luciferase activity was measured using Dual-Luciferase^®^ Reporter Assay System. Firefly luciferase activity was normalized by pRL-SV40 *Renilla* luciferase activity. Each bar represents mean ± S.E.M. from three independent experiments carried out in quadruplicate. *P* value, Student’s t-test.

The effect of the rs2274971A > G polymorphisms on the promoter activity of *ENO1* was investigated in a luciferase assay. For this analysis, we generated pGL3-Basic-*ENO1* constructs containing rs2274971A > G and transfected the constructs into NSCLC cells (A549, H1299, and H1703). The rs2274971G allele exhibited significantly higher promoter activity than the rs2274971A allele (*P* = 3 × 10^−4^, *P* = 7 × 10^−9^, and *P* = 2 × 10^−5^, respectively, Fig. 2A). Because *ENO1* expression is known to be induced by hypoxia and the SNP site in the fragment cloned into pGL3-basic vector is predicted to lie in a HIF-1α binding site according to SNPinfo website (https://snpinfo.niehs.nih.gov/cgi-bin/snpinfo/tfbs.cgi? 2_rs2274971), we further performed promoter assay under hypoxia and HIF-1α overexpression. For HIF-1α overexpression, we cotransfected pGL3-Basic-*ENO1* constructs into A549 cells with pEGFP-HIF-1α. The luciferase activity was significantly increased by hypoxia and HIF-1α overexpression (Fig. 2B,C). Under hypoxia and HIF-1α overexpression, promoter activity was consistently higher in rs2274971G allele than the rs2274971A allele (*P* = 7 × 10^−4^, Fig. 2B, and *P* = 1 × 10^−4^, Fig. 2C). These results suggest that the rs2274971G allele is associated with higher expression of *ENO1* compared with rs2274971A allele and that hypoxia and HIF-1α upregulate *ENO1* expression.

## Discussion

This study was conducted to investigate whether genetic polymorphisms in the glycolytic pathway affect the prognosis of patients with NSCLC after surgical resection. Among the 44 SNPs evaluated, the *ENO1* rs2274971A > G, *PFKM* rs11168417C > T, *PFKP* rs1132173C > T, and *PDK2* rs3785921G > A polymorphisms were significantly associated with survival outcomes. More importantly, combined analysis of the four SNPs more precisely predicted prognosis. In addition, stratified analysis by tumor histology suggested that genetic polymorphisms in the glycolytic pathway exert a different prognostic effect according to tumor histology of NSCLC. These results suggest that the glycolytic pathway plays an important role in determining the prognosis of early stage NSCLC. SNP analysis of glycolytic genes may be useful for predicting prognosis after surgical resection in NSCLC, thereby help to refine therapeutic decisions.

In the present study, *ENO1* rs2274971A > G, *PFKM* rs11168417C > T, *PFKP* rs1132173C > T, and *PDK2* rs3785921G > A polymorphisms in glycolytic pathway were associated with survival outcomes after surgery, suggesting that the SNPs in glycolytic pathway may be prognostic markers in early stage NSCLC. However, because glycolysis involves sequential reactions mediated by a series of enzymes interacting in the glycolytic process, it is unlikely that the prognosis of lung cancer patients can be determined by a single genetic polymorphism in the glycolytic pathway. Instead, a pathway-based multigenic approach may amplify the effects of individual polymorphisms and have better resolution in predicting prognosis^12^,^13^. Although four SNPs were associated with survival outcomes in individual SNP analysis, considering the borderline CI and multiple comparisons issue, the impact of any individual SNP on survival outcomes may be marginal. When the adverse genotypes of four SNPs were combined, these genotypes additively contributed to decreased survival, enabling more precise prediction of prognosis. Our results suggest that combined analysis of multiple SNPs in relevant pathways has a higher potential value for identifying SNPs with prognostic significance.

Enolase (2-phospho-D-glycerate hydrolase) is one of the key glycolytic enzymes that catalyzes the reversible conversion of 2-phosphoglycerate to phophoenolpyruvate. The non-neuronal enolase (α-enolase, ENO1) plays an important role in several physiological processes depending on the cellular localization^14^. ENO1 is mainly localized in the cytoplasm to participate in glycolysis. In addition to its glycolytic enzyme activity, it is expressed on the cell surface and acts as a plasminogen receptor to bind and activate plasminogen and promote degradation of the extracellular matrix for cell migration and cancer metastasis^15^. In contrast, *ENO1* can be alternatively translated into a c-Myc promoter binding protein, which is localized to the nucleus to bind to the *c-Myc* promoter and suppress the transcription of *c-Myc*^14^,^16^, suggesting a tumor suppressor function. Overexpression of ENO1 has been correlated with tumor progression and poor prognosis in many tumor tissues^17^,^18^, whereas its down-regulation has also been reported and associated with poor prognosis in several cancers^16^,^19^. In the present study, the *ENO1* rs2274971G allele, which exhibited significantly higher promoter activity compared to the rs2274971A allele, was associated with better survival outcomes, suggesting that ENO1 has a potential tumor suppressor function. However, *ENO1* rs2274971A > G was not significantly associated with clinicopathological features such as age, sex, smoking status, diabetes, tumor histology, pathologic stage, and histologic grade (data not shown).

A key step in glycolysis, the phosphorylation of fructose 6-phosphate to fructose 1, 6 bisphosphate, is catalyzed by phosphofructokinase-1 (PFK-1) and commits glucose to the glycolytic pathway. PFK-1 activity is rate-limiting, critical in determining glycolytic flux, and tightly regulated^20^,^21^. PFK-1 is a tetramer composed of three types of subunits, M (muscle), L (liver), and P (platelet), which are encoded by three different genes, *PFKM*, *PFKL*, and *PFKP*, respectively. PFK-1 tetrameric isoenzymes have various subunit compositions, and thus different kinetic properties and activities, according to the tissue type^22^. PFK-1 activity is increased in response to proliferation signals and is correlated with elevated glycolysis in proliferating cells^21^. Elevated PFK-1 activity is induced by oncogenic signals or HIF-1α activation, which is characteristic of cancer cells^21^,^23^. PDK phosphorylates and inhibits pyruvate dehydrogenase, preventing pyruvate from entering mitochondrial matrix to form acetyl-CoA for the Krebs cycle. Increased expression or activity of PDK may play a role in the metabolic shift away from mitochondrial oxidation to facilitate the glycolytic process in cancer^24^,^25^. PDK is frequently upregulated in tumor tissues by MYC, HIF activation, or TP53 loss^25^,^26^,^27^. PDK2 is the most widely distributed and best characterized isoform among the four PDK isoforms^28^. Particularly, PDK2 is the most sensitive to dichloroacetic acid, which activates pyruvate dehydrogenase by inhibiting PDK and shifts the metabolism of cancer cells from glycolysis to oxidative phosphorylation. Dichloroacetic acid has been shown to have anti-cancer effect in preclinical studies^29^,^30^, and has been tested in early clinical trials in several cancers^31^,^32^. *PFKP* rs1132173 (Phe309Phe) is a synonymous SNP, and *PFKM* rs11168417 and *PDK2* rs3785921 are intronic variants. Although synonymous SNPs and intronic SNPs have been considered non-functional, studies have reported that synonymous variations may affect protein translation efficiency, structure, and function, as well as splicing events, mRNA stability, and microRNA binding^33^,^34^. Recent results of the Encyclopedia of DNA Elements (ENCODE) project provided evidence that genetic variations in non-coding DNA, such as intronic SNP, play an important role in the regulation of gene expression^35^. An alternative explanation is that the association between the SNPs and survival outcomes may be due to linkage disequilibrium (LD) with other functional variants. Future studies are needed to understand the biologic mechanism of the observed associations between the SNPs and survival outcomes.

Interestingly, stratified analysis by tumor histology suggested that polymorphisms in glycolytic pathway genes may play a different prognostic role according to the tumor histology of NSCLC. This finding is comparable to those of previous studies^36^,^37^ showing that glucose metabolism differs between SCC and AC of the lung: SCC was associated with higher ^18^F-FDG uptake and higher levels of glycolysis-related markers compared to AC. Further studies are required to understand the role of glycolysis in the pathogenesis and prognosis of NSCLC, particularly the differential role in SCC and AC. There were several limitations to this study. First, this study did not provide direct evidence that the four genes are involved in the development and progression of lung cancer, limiting the applicability to the biological mechanism of the observed associations between the SNPs and survival outcomes. Second, all patients were treated at the same hospital, which may have led to a selection bias. Third, this study included only a Korean patient population, and thus the results may not be generalizable for other ethnic groups.

In conclusion, the analysis of glycolytic pathway gene polymorphisms, particularly combined analysis, may be useful for predicting patient prognosis after surgery, thereby helping to refine therapeutic decisions in NSCLC. Future studies are warranted to understand the biological mechanism of our findings and validate our results in a larger number of patients, including diverse ethnic groups.

## Materials and Methods

### Study populations

A total of 782 patients with pathologic stages I, II, or IIIA (micro-invasive N2) NSCLC who underwent curative surgical resection at Kyungpook National University Hospital (KNUH, n = 354) and Seoul National University Bundang Hospital (SNUBH, n = 428) were enrolled in this study. All patients included in this study were ethnic Koreans. None of the patients received chemotherapy or radiotherapy prior to surgery. The pathologic staging of tumors was determined according to the International System for Staging Lung Cancer^2^. Written informed consent was obtained from all patients prior to surgery at each of the participating institutions. This study was approved by the institutional review boards of KNUH and SNUBH, and was performed in accordance with the research protocol which was approved by the institutional review boards of KNUH and SNUBH.

### Selection of SNPs and genotyping

To collect potentially functional polymorphisms in glycolytic pathway genes, we first searched the public SNP database (http://www.ncbi.nlm.nih.gov/SNP) for all SNPs in major glycolytic genes. Next, using the FuncPred utility for functional SNP prediction and TagSNP utility for LD tag SNP selection in the SNPinfo web server (https://snpinfo.niehs.nih.gov/), a total of 54 potentially functional SNPs in 19 glycolytic pathway genes with minor allele frequency ≥ 0.05 in the HapMap JPT data were collected after excluding those in LD (*r*^2^ ≥ 0.8). Genomic DNA was extracted from peripheral blood lymphocytes using blood QuickGene DNA whole blood kit S (Fujifilm, Tokyo, Japan). Genotyping was performed using the MassARRAY^®^ iPLEX assay (SEQUENOM Inc., San Diego, CA, USA). Duplicate samples and negative controls were included to ensure the accuracy of genotyping. Approximately 5% of the samples were randomly selected to be genotyped again with a restriction fragment length polymorphism assay by a different investigator and the results were 100% concordant.

### Promoter-luciferase constructs and luciferase assay

We investigated whether rs2274971A > G ( + 487 from transcription start site) of *ENO1* coding alpha-enolase modulates the promoter activity of the gene by conducting a luciferase assay. A 1151-bp fragment (from −387 to + 764) including rs2274971A > G was synthesized by polymerase chain reaction using genomic DNA. The forward primer with a KpnI restriction site (5′-GGGGTACCAGTGGTGCTTCAACTGGTATC-3′) and reverse primer with a XhoI restriction site (5′-CCGCTCGAGTCAATCAGTTACCTGAGTGC-3′) were used. The polymerase chain reaction products were cloned into the KpnI/XhoI sites of the pGL3-basic vector (Promega, Madison, WI, USA), resulting in pGL3-Basic-*ENO1* constructs containing rs2274971A > G. The correct sequence of all clones was verified by DNA sequencing. Human NSCLC cells (A549, H1299, and H1703) were purchased from Korean Cell Line Bank (KCLB), Seoul, Korea, and authenticated by KCLB using short tandem repeat DNA fingerprinting. The cells were transfected with pRL-SV40 vector (Promega) and pGL3-basic vector using Effectene^®^ transfection reagent (Qiagen, Hilden, Germany). The cells were harvested 24 h after transfection and lysates were prepared using a Dual-Luciferase^®^ Reporter Assay System (Promega). Luciferase activity was measured using a Synergy^TM^ HTX Multi-Mode Microplate Reader (BioTek Instruments, Winooski, VT, USA). The results were normalized to those of pRL-SV40 *Renilla* luciferase activity. All experiments were performed three times in quadruplicate.

### Hypoxia condition and HIF-1α overexpression

At 24 h posttransfection with pRL-SV40 and pGL3-basic-*ENO1* containing rs2274971A or G allele, A549 cells were exposed to hypoxia for 24 h. For hypoxia, the cells were incubated at 5% CO_2_, 1% O_2_, 94% N_2_ in a hypoxic chamber (Invivo_2_ 400, Ruskinn Technologies, UK) at 37 °C. For HIF-1α overexpression, A549 cells were transfected with pRL-SV40, pGL3-basic-*ENO1* containing rs2274971A or G allele, and pEGFP-HIF-1α.

### Western blot analysis

Cells were harvested using ProNA^TM^ CETi Lysis Buffer containing protease inhibitors and phosphatase inhibitors (Translab, Daejeon, Korea), and protein concentration was determined using the Pierce^TM^ BCA Protein Assay Kit (Thermo Fisher Scientific, Waltham, MA, USA). Protein samples were resolved by SDS-PAGE and transferred to a pre-wetted polyvinylidene difluoride membranes (Millipore Corporation, Bedford, MA, USA). The membrane was incubated in blocking buffer. Anti-HIF-1α (Novus Biologicals, Littleton, CO, USA) primary antibody was used. The primary β-actin (Santa Cruz Biotech, Santa Cruz, CA, USA) was used as an internal control. Immunoreactive proteins were visualized by SuperSignal™ West Femto Maximum Sensitivity Substrate kit (Thermo Fisher Scientific, Waltham, MA, USA).

### Statistical analysis

Differences in the distribution of genotypes according to the clinicopathologic factors of patients were compared using χ^2^ tests. OS was measured from the day of surgery until the date of death or to the last follow-up date. DFS was estimated from the day of surgery until recurrence or death. The survival estimates were calculated by the Kaplan-Meier method. The differences in OS and DFS across different genotypes were compared by the log-rank test. HRs and 95% CIs were estimated using multivariate Cox proportional hazards models, with adjustment for age, gender, smoking status, tumor histology, pathologic stage, and adjuvant therapy. All analyses were performed with Statistical Analysis System for Windows, version 9.2 (SAS Institute, Cary, NC, USA).

## Additional Information

**How to cite this article**: Lee, S. Y. *et al*. Genetic Polymorphisms in glycolytic pathway are associated with the prognosis of patients with early stage non-small cell lung cancer. *Sci. Rep.* **6**, 35603; doi: 10.1038/srep35603 (2016).

## Supplementary Material

Supplementary Information

## Figures and Tables

**Table 1:** Univariate analysis for survival outcomes by clinicopathological features.

Variables	No. of cases	Overall survival			Disease free survival		
		No. of deaths (%)^a^	5Y-OSR (%)^b^	Log-rank *P*	No. of events (%)^a^	5Y-DFSR (%)^b^	Log-rank *P*
Overall	782	208 (26.6)	62		340 (43.5)	45	
Age (years)							
< 65	383	88 (23.0)	69	2 × 10^−3^	162 (42.3)	48	0.14
≥ 65	399	120 (30.1)	55		178 (44.6)	41	
Sex							
Male	572	173 (30.2)	59	4 × 10^−4^	261 (45.6)	42	0.10
Female	210	35 (16.7)	71		79 (37.6)	52	
Smoking status							
Never	232	40 (17.2)	74	3 × 10^−4^	90 (38.8)	50	0.15
Ever	550	168 (30.6)	57		250 (45.5)	43	
Diabetes							
No	619	150 (24.2)	66	0.03	259 (41.8)	47	0.19
Yes	152	51 (33.6)	51		73 (48.0)	36	
Histological types							
SCC	341	103 (30.2)	60	0.17	146 (42.8)	48	0.22
AC	425	99 (23.3)	63		184 (43.3)	42	
LCC	16	6 (37.5)	59		10 (62.5)	35	
Pathologic stage							
I	378	59 (15.6)	76	1 × 10^−11^	107 (28.3)	60	2 × 10^−^1^5^
II	227	81 (35.7)	52		116 (51.1)	39	
IIIA	177	68 (38.4)	47		117 (66.1)	20	
Adjuvant therapy^c^							
No	184	72 (39.6)	49	0.58	102 (56.0)	37	0.36
Yes	220	77 (34.7)	50		131 (59.0)	25	
Body mass index							
< 25	464	107 (23.1)	65		200 (43.1)	41	
≥ 25	183	45 (24.6)	61	0.85	75 (41.0)	46	0.40

Abbreviations: 5Y-OSR, five year-overall survival rate; 5Y-DFSR, five year-disease free survival rate; SCC, squamous cell carcinoma; AC, adenocarcinoma; LCC, large cell carcinoma.

^a^Row percentage.

^b^Five year-overall survival rate and five year-disease free survival rate, proportion of survival derived from Kaplan-Meier analysis.

^c^In pathologic stages II + IIIA: 182 cases received adjuvant chemotherapy alone, 11 cases received adjuvant radiotherapy alone, and 27 cases received both chemotherapy and radiotherapy.

**Table 2:** Overall survival and disease free survival according to the *ENO1* rs2274971A > G, *PFKM* rs11168417C > T, *PFKP* rs1132173C > T, and *PDK2* rs3785921G > A genotypes.

Polymorphism/genotype	No. of Cases (%)^a^	Overall survival				Disease free survival			
		No. of deaths (%)^b^	5Y-OSR (%)^c^	HR (95% CI)^d^	*P*^d^	No. of Event (%)^b^	5Y-DFSR (%)^c^	HR (95% CI)^d^	*P*^d^
*ENO1* rs2274971^e^									
AA	448 (59.1)	128 (28.6)	58	1.00		205 (45.8)	41	1.00	
AG	266 (35.1)	63 (23.7)	67	0.73 (0.54–1.00)	0.05	105 (39.5)	50	0.73 (0.58–0.93)	0.01
GG	44 (5.8)	8 (18.2)	76	0.55 (0.27–1.12)	0.10	15 (34.10	59	0.69 (0.41–1.17)	0.17
Dominant	310 (40.9)	71 (22.9)	68	0.70 (0.52–0.95)	0.02	120 (38.7)	51	0.73 (0.58–0.92)	7 × 10^−3^
Recessive	714 (94.2)	191 (26.8)	61	0.61 (0.30–1.24)	0.17	310 (43.4)	44	0.78 (0.46–1.31)	0.34
Codominant				0.73 (0.57–0.94)	0.02			0.77 (0.64–0.94)	0.01
*PFKM* rs11168417^e^									
CC	471 (61.1)	109 (23.1)	67	1.00		186 (39.5)	49	1.00	
CT	264 (34.2)	88 (33.3)	53	1.49 (1.11–1.99)	8 × 10^−3^	132 (50.0)	40	1.28 (1.02–1.61)	0.04
TT	36 (4.7)	8 (22.2)	62	1.11 (0.54–2.29)	0.78	14 (38.9)	38	1.03 (0.59–1.77)	0.93
Dominant	300 ((38.9)	96 (32.0)	54	1.44 (1.09–1.91)	0.01	146 (48.7)	39	1.25 (1.00–1.56)	0.05
Recessive	735 (95.3)	197 (26.8)	62	0.95 (0.47–1.93)	0.89	318 (43.3)	46	0.94 (0.55–1.60)	0.81
Codominant				1.28 (1.02–1.61)	0.04			1.15 (0.96–1.38)	0.13
*PFKP* rs1132173^e^									
CC	207 (26.9)	57 (27.5)	65	1.00		90 (43.5)	46	1.00	
CT	401 (52.1)	113 (28.2)	57	1.06 (0.76–1.48)	0.72	184 (45.9)	41	1.09 (0.84–1.42)	0.50
TT	162 (21.0)	32 (19.8)	73	0.83 (0.53–1.29)	0.41	57 (35.2)	55	0.77 (0.55–1.08)	0.14
Dominant	563 (73.1)	145 (25.8)	62	1.00 (0.73–1.37)	0.99	241 (42.8)	45	0.99 (0.78–1.27)	0.96
Recessive	608 (79.0)	170 (28.0)	60	0.80 (0.54–1.17)	0.25	274 (45.1)	43	0.73 (0.55–0.98)	0.03
Codominant				0.93 (0.76–1.15)	0.49			0.90 (0.77–1.06)	0.20
*PDK2* rs3785921^e^									
GG	342 (44.3)	85 (24.9)	64	1.00		133 (38.9)	49	1.00	
GA	356 (46.1)	95 (26.7)	62	1.31 (0.97–1.76)	0.08	163 (45.8)	42	1.39 (1.10–1.76)	5 × 10^−3^
AA	74 (9.6)	23 (31.1)	59	1.34 (0.82–2.17)	0.24	36 (48.7)	40	1.44 (0.98–2.11)	0.06
Dominant	430 (55.7)	118 (27.4)	62	1.31 (0.99–1.75)	0.06	199 (46.3)	42	1.40 (1.12–1.75)	3 × 10^−3^
Recessive	698 (90.4)	180 (25.8)	63	1.17 (0.74–1.85)	0.50	296 (42.4)	46	1.22 (0.85–1.74)	0.29
Codominant				1.20 (0.98–1.49)	0.08			1.26 (1.07–1.49)	6 × 10^−^3

Abbreviations: 5Y-OSR, five year-overall survival rate; 5Y-DFSR, five year-disease free survival rate; HR, hazard ratio; CI, confidence interval.

^a^Column percentage.

^b^Row percentage.

^c^Five year-overall survival rate and five year-disease free survival rate, proportion of survival derived from Kaplan-Meier analysis.

^d^HRs, 95% CIs, and their corresponding *P* values were calculated using multivariate Cox proportional hazard models, adjusted for age, gender, smoking status, diabetes, tumor histology, pathologic stage, adjuvant therapy, and body mass index.

^e^Genotype failures: 24 case for rs2274971, 11 case for rs11168417, 12 case for rs1132173, and 10 case for rs3785921.

**Table 3:** Overall survival and disease free survival according to the *ENO1* rs2274971A > G, *PFKM* rs11168417C > T, *PFKP* rs1132173C > T, and *PDK2* rs3785921G > A genotypes stratified by tumor histology.

Polymorphism/genotype	Overall survival				*P*_H_^b^	Disease free survival				*P*_H_^b^
	SCC		AC			SCC		AC		
	HR (95% CI)^a^	*P*^a^	HR (95% CI)^a^	*P*^a^		HR (95% CI)^a^	*P*^a^	HR (95% CI)^a^	*P*^a^	
*ENO1* rs2274971										
Dominant	0.43 (0.26–0.70)	6 × 10^−4^	1.10 (0.73–1.65)	0.65	4 × 10^−3^	0.53 (0.36–0.78)	1 × 10^−3^	0.95 (0.70–1.28)	0.73	0.02
Recessive	0.58 (0.21–1.60)	0.29	0.65 (0.24–1.79)	0.41	0.88	0.84 (0.39–1.81)	0.65	0.77 (0.38–1.57)	0.47	0.87
Codominant	0.52 (0.34–0.78)	2 × 10^−3^	1.01 (0.72–1.40)	0.97	0.01	0.62 (0.45–0.87)	5 × 10^−3^	0.93 (0.72–1.20)	0.57	0.06
*PFKM* rs11168417										
Dominant	1.82 (1.22–2.72)	4 × 10^−3^	1.15 (0.76–1.74)	0.50	0.12	1.90 (1.35–2.67)	2 × 10^−4^	0.96 (0.71–1.30)	0.78	3 × 10^−3^
Recessive	1.14 (0.46–2.83)	0.77	0.54 (0.13–2.21)	0.39	0.38	0.89 (0.39–2.02)	0.77	0.90 (0.42–1.95)	0.80	0.98
Codominant	1.50 (1.10–2.06)	0.01	1.05 (0.74–1.51)	0.78	0.15	1.48 (1.14–1.91)	3 × 10^−3^	0.96 (0.74–1.24)	0.75	0.02
*PFKP* rs1132173										
Dominant	1.00 (0.63–1.57)	0.98	0.97 (0.61–1.54)	0.90	0.93	1.01 (0.69–1.48)	0.98	0.93 (0.66–1.30)	0.67	0.75
Recessive	1.25 (0.74–2.10)	0.41	0.48 (0.26–0.89)	0.02	0.02	0.96 (0.62–1.50)	0.87	0.57 (0.38–0.85)	6 × 10^−3^	0.09
Codominant	1.07 (0.79–1.46)	0.65	0.80 (0.59–1.07)	0.14	0.18	0.99 (0.77–1.27)	0.94	0.81 (0.66–1.00)	0.05	0.23
*PDK2* rs3785921										
Dominant	1.32 (0.88–2.00)	0.18	1.23 (0.81–1.86)	0.34	0.81	1.59 (1.12–2.26)	9 × 10^−3^	1.19 (0.87–1.61)	0.27	0.22
Recessive	1.58 (0.88–2.86)	0.13	0.88 (0.42–1.83)	0.72	0.22	1.89 (1.15–3.10)	0.01	0.78 (0.45–1.36)	0.38	0.02
Codominant	1.30 (0.96–1.74)	0.09	1.09 (0.80–1.49)	0.57	0.42	1.49 (1.16–1.91)	2 × 10^−3^	1.05 (0.84–1.32)	0.67	0.04

Abbreviations: SCC, squamous cell carcinoma; AC, adenocarcinoma; HR, hazard ratio; CI, confidence interval.

^a^HRs, 95% CIs, and their corresponding *P* values were calculated using multivariate Cox proportional hazard models, adjusted for age, gender, smoking status, diabetes, pathologic stage, adjuvant therapy, and body mass index.

^b^*P*_H_, *P* for heterogeneity test.

**Table 4:** Combined effects of the SNPs on overall survival and disease free survival.

No. of bad genotypes^a^	No. of cases (%)^b^	Overall survival					Disease free survival				
		No. of deaths (%)^c^	5Y-OSR (%)^d^	*P*_(L-R)_	HR (95% CI)^e^	*P*^e^	No. of events (%)^c^	5Y-DFSR (%)^d^	*P*_(L-R)_	HR (95% CI)^e^	*P*^e^
All cases^f^											
0 or 1^g^	145 (19.5)^f^	20 (13.8)	79	5 × 10^−4^	1.00		38 (26.2)	63	6 × 10^−7^	1.00	
2	279 (37.6)	78 (28.0)	60		2.26 (1.36–3.74)	2 × 10^−3^	126 (45.2)	44		2.02 (1.40–2.93)	2 × 10^−4^
3	231 (31.1)	68 (29.4)	58		2.59 (1.55–4.32)	3 × 10^−4^	107 (46.3)	40		2.35 (1.62–3.43)	8 × 10^−6^
4	88 (11.8)	27 (30.7)	53		2.62 (1.46–4.70)	1 × 10^−3^	45 (51.1)	38		2.37 (1.54–3.67)	1 × 10^−4^
*P*_trend_						8 × 10^−4^					3 × 10^−5^
0 + 1^g^	145 (19.5)^f^	20 (13.8)	79	3 × 10^−5^	1.00		38 (26.2)	63	9 × 10^−7^	1.00	
2 + 3 + 4	598 (80.5)	173 (29.0)	58		2.44 (1.51–3.92)	3 × 10^−4^	278 (46.5)	41		2.20 (1.56–3.10)	8 × 10^−6^
SCC^h^											
0	41 (12.4)	4 (9.8)	86	2 × 10^−3^	1.00		7 (17.1)	74	3 × 10^−6^	1.00	
1	120 (36.3)	31 (25.8)	66		3.28 (0.99–10.87)	0.05	45 (37.5)	54		3.04 (1.28–7.21)	0.01
2	116 (35.1)	40 (34.5)	52		5.30 (1.62–17.37)	6 × 10^−3^	56 (48.3)	38		4.56 (1.94–10.73)	5 × 10^−4^
3	54 (16.3)	24 (44.4)	46		7.67 (2.28–25.80)	1 × 10^−3^	32 (59.3)	37		6.76 (2.77–16.50)	3 × 10^−5^
*P*_trend_						2 × 10^−5^					5 × 10^−7^

Abbreviations: 5Y-OSR, five year-overall survival rate; 5Y-DFSR, five year-disease free survival rate; HR, hazard ratio; CI, confidence interval; *P*_L-R,_ Log-rank *P*; SCC, squamous cell carcinoma; AC, adenocarcinoma.

^a^*ENO1* rs2274971 AA, *PFKM* rs11168417 CT + TT, *PFKP* rs1132173 CC + CT, and *PDK2* rs3785921 GA + AA.

^b^Column percentage.

^c^Row percentage.

^d^Five year-overall survival rate and five year-disease free survival rate, proportion of survival derived from Kaplan-Meier analysis.

^e^HRs, 95% CIs, and their corresponding *P* values were calculated using multivariate Cox proportional hazard models, adjusted for age, gender, smoking status, diabetes, tumor histology, pathologic stage, adjuvant therapy, and body mass index.

^f^Combined genotypes of rs11168417, rs2274971, rs3785921, rs1132173. Thirty nine patients with missing genotype data at any of the four SNPs.

^g^21 cases for zero bad genotype and 124 cases for one bad genotype. Because zero bad genotype had no death, zero and one bad genotypes were combined.

^h^Combined genotypes of rs11168417, rs2274971, rs3785921. Ten patients with missing genotype data at any of the three SNPs.
