# Supplementary material for: Genetic polymorphisms in glycolytic pathway are associated with the prognosis of patients with early stage non-small cell lung cancer
Source: Sci Rep. 2016 Oct 21;6:35603. doi: 10.1038/srep35603 (PMC5073284; doi:10.1038/srep35603)
Supplement: Supplementary Information [file srep35603-s1.pdf]

Genetic polymorphisms in glycolytic pathway are associated with the prognosis of patients with early stage non-small cell lung cancer

Shin Yup Lee[1,2,*], Cheng Cheng Jin[3,4,*], Jin Eun Choi[3,5], Mi Jeong Hong[3,4], Deuk Kju Jung[3,4], Sook Kyung Do[3,4], Sun Ah Baek[3], Hyo Jung Kang[3], Hyo-Gyoung Kang[3,5], Sun Ha Choi[2], Won Kee Lee[6], Yangki Seok[1,7], Eung Bae Lee[1,7], Ji Yun Jeong[8], Kyung Min Shin[9], Sukki Cho[10], Seung Soo Yoo[1,2], Jaehee Lee[2], Seung Ick Cha[2], Chang Ho Kim[2], You Mie Lee[11], In-Kyu Lee[2], Sanghoon Jheon[10] & Jae Yong Park[1,2,3,4,5]

[1]Lung Cancer Center, Kyungpook National University Medical Center, Daegu 41404, Republic of Korea. [2]Departments of Internal Medicine, School of Medicine, Kyungpook National University, Daegu 41944, Republic of Korea. [3]Department of Biochemistry and Cell Biology, School of Medicine, Kyungpook National University, Daegu 41944, Republic of Korea. [4]BK21 Plus KNU Biomedical Convergence Program, Department of Biomedical Science, Kyungpook National University, Daegu 41944, Republic of Korea. [5]Cell and Matrix Research Institute, School of Medicine, Kyungpook National University, Daegu 41944, Republic of Korea. [6]Biostatistics Center, School of Medicine, Kyungpook National University, Daegu 41944, Republic of Korea. [7]Department of Thoracic Surgery, School of Medicine, Kyungpook National University, Daegu 41944, Republic of Korea. [8]Department of Pathology, School of Medicine, Kyungpook National University, Daegu 41944, Republic of Korea. [9]Department of Radiology, School of Medicine, Kyungpook National University, Daegu 41944, Republic of Korea. [10]Department of Thoracic and Cardiovascular Surgery, Seoul National University School of Medicine, Seoul 13620, Republic of Korea, [11]College of Pharmacy, Kyungpook National University, Daegu, 41566, Republic of Korea;

*These authors contributed equally to this work. Correspondence and requests for materials should be addressed to S.Y.L. (email: shinyup@knu.ac.kr) or J.Y.P. (email: jaeyong@knu.ac.kr)

Supplementary Table 1. List of analyzed SNPs and the association with survival outcomes.

				P^a for Overall survival			P^a for Disease-free survival		
SNP ID	Gene	Base change	MAF	Dominant	Recessive	Codominant	Dominant	Recessive	Codominant
rs2274971	ENO1	A>G	0.23	0.02	0.16	0.02	0.01	0.40	0.02
rs11168417	PFKM	C>T	0.22	0.02	0.82	0.05	0.04	0.76	0.10
rs1132173	PFKP	C>T	0.47	0.72	0.11	0.24	0.74	0.02	0.12
rs3785921	PDK2	G>A	0.33	0.10	0.39	0.10	0.003	0.22	0.01
rs7971637	GAPDH	C>T	0.28	0.17	0.05	0.05	0.24	0.10	0.10
rs2986408	PDHX	A>C	0.15	0.35	0.31	0.26	0.43	0.10	0.23
rs2071064	TPI1	C>T	0.30	0.08	0.51	0.30	0.44	0.87	0.61
rs8192431	PKM	C>T	0.36	0.83	0.31	0.72	0.56	0.08	0.64
rs4880592	PFKP	G>A	0.47	0.63	0.56	0.98	0.80	0.09	0.25
rs656489	HK2	G>A	0.30	0.29	0.48	0.65	0.19	0.08	0.87
rs7231	PDHB	G>C	0.24	0.56	0.11	0.25	0.76	0.10	0.35
rs17032801	ENO1	A>G	0.42	0.25	0.41	0.75	0.50	0.25	0.84
rs1057034	PFKL	C>T	0.36	0.52	0.30	0.33	0.55	0.16	0.25
rs4757650	LDHA	C>T	0.09	0.29	0.72	0.35	0.47	0.77	0.54
rs8112853	GAPDHS	G>C	0.27	0.56	0.63	0.51	0.19	0.26	0.13
rs1053000	PFKP	C>T	0.23	0.92	0.42	0.79	0.96	0.46	0.73
rs6489721	GAPDH	T>C	0.39	0.50	0.10	0.68	0.51	0.16	0.76
rs7100037	PFKP	T>C	0.36	0.55	0.63	0.51	0.19	0.69	0.26
rs12302749	TPI1	C>T	0.21	0.60	0.43	0.48	0.41	0.73	0.54
rs2306302	PFKP	G>A	0.09	0.72	0.22	0.54	0.48	0.64	0.59
rs4908519	ENO1	C>T	0.16	0.74	0.22	0.53	0.20	0.34	0.35
rs1803621	GAPDH	C>T	0.49	0.37	0.35	0.98	0.35	0.51	0.86
rs7128549	LDHA	C>G	0.36	0.46	0.48	0.86	0.17	0.52	0.50
rs7176223	ADPGK	T>G	0.24	0.83	0.92	0.83	0.54	0.66	0.73
rs4806173	GAPDHS	C>G	0.29	0.46	0.63	0.43	1.00	0.58	0.82
rs10492080	PFKM	C>T	0.46	0.38	0.22	0.20	0.46	0.12	0.16
rs2956107	PDHX	C>T	0.27	0.17	0.32	0.14	0.50	0.21	0.29
rs11064436	TPI1	G>C	0.19	0.35	0.29	0.25	0.80	0.56	0.69
rs10181851	PDK1	A>G	0.21	0.61	0.20	0.38	0.38	0.24	0.25
rs7248411	GPI	C>G	0.18	0.72	0.60	0.63	0.50	0.72	0.64
rs2229626	HK2	C>T	0.46	0.53	0.58	0.47	0.34	0.92	0.50
rs4760619	PFKM	A>T	0.23	0.75	0.35	0.55	0.66	0.57	0.85
rs1052309	PFKP	G>A	0.19	0.38	0.33	0.30	0.97	0.96	0.96
rs608112	HK2	G>A	0.23	0.57	0.62	0.79	0.37	0.26	0.79
rs4806174	GAPDHS	G>C	0.37	0.33	0.80	0.41	0.68	0.50	0.96

Supplementary Table 1. Continued.

SNP ID	Gene	Base change	MAF	P^a for Overall survival			P^a for Disease-free survival		
				Dominant	Recessive	Codominant	Dominant	Recessive	Codominant
rs2073436	PFKL	C>G	0.21	0.95	0.80	0.97	0.84	0.76	0.79
rs12276735	PDHX	G>A	0.11	0.18	0.98	0.35	0.84	0.71	0.92
rs3807891	PDK4	C>G	0.30	0.62	0.17	0.32	0.62	0.73	0.60
rs2001004	TPI1	A>G	0.46	0.30	0.94	0.49	0.38	0.79	0.48
rs3732298	HK2	T>C	0.12	0.57	0.14	0.33	0.64	0.20	0.95
rs8040828	PKM	C>A	0.39	0.57	0.75	0.83	0.22	0.70	0.31
rs8023358	ADPGK	A>G	0.22	0.65	0.25	0.95	0.61	0.13	0.33
rs1346165	ADPGK	C>T	0.21	0.96	0.39	0.78	0.41	0.17	0.24
rs2278543	ADPGK	G>A	0.46	0.58	0.62	0.95	0.63	0.65	0.57

MAF, minor allele frequency

[a] *P*-values calculated using multivariate Cox proportional hazard models, adjusted for age, gender, smoking status, tumor histology, pathologic stage, and adjuvant therapy.

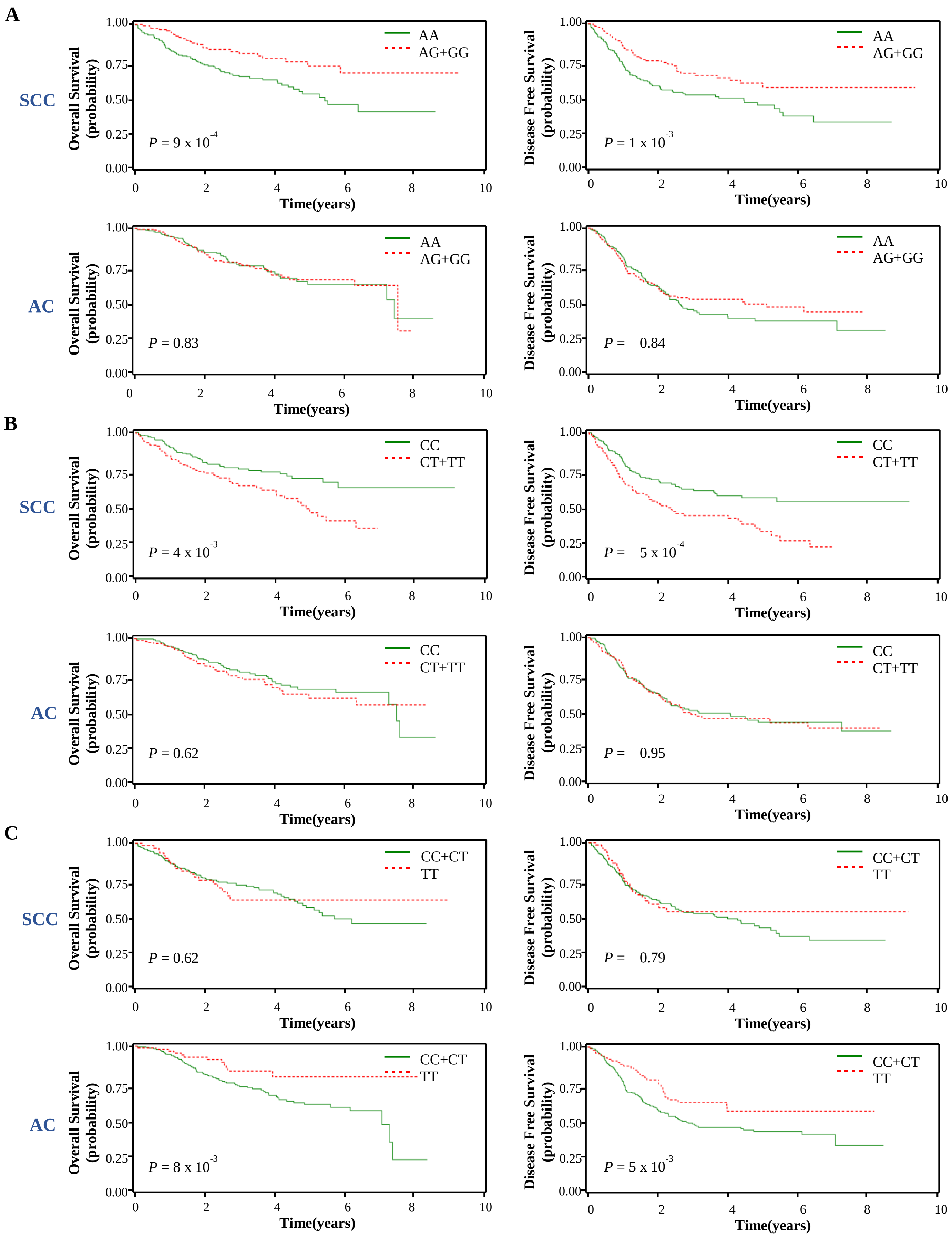
A
SCC
Overall Survival (probability)
Time(years)
AA
AG+GG
P = 9 x 10-4
Disease Free Survival (probability)
AA
AG+GG
P = 1 x 10-3
AC
AA
AG+GG
P = 0.83
AA
AG+GG
P = 0.84
B
SCC
CC
CT+TT
P = 4 x 10-3
CC
CT+TT
P = 5 x 10-4
AC
CC
CT+TT
P = 0.62
CC
CT+TT
P = 0.95
C
SCC
CC+CT
TT
P = 0.62
CC+CT
TT
P = 0.79
AC
CC+CT
TT
P = 8 x 10-3
CC+CT
TT
P = 5 x 10-3
0.00
0.25
0.50
0.75
1.00
0
2
4
6
8
10

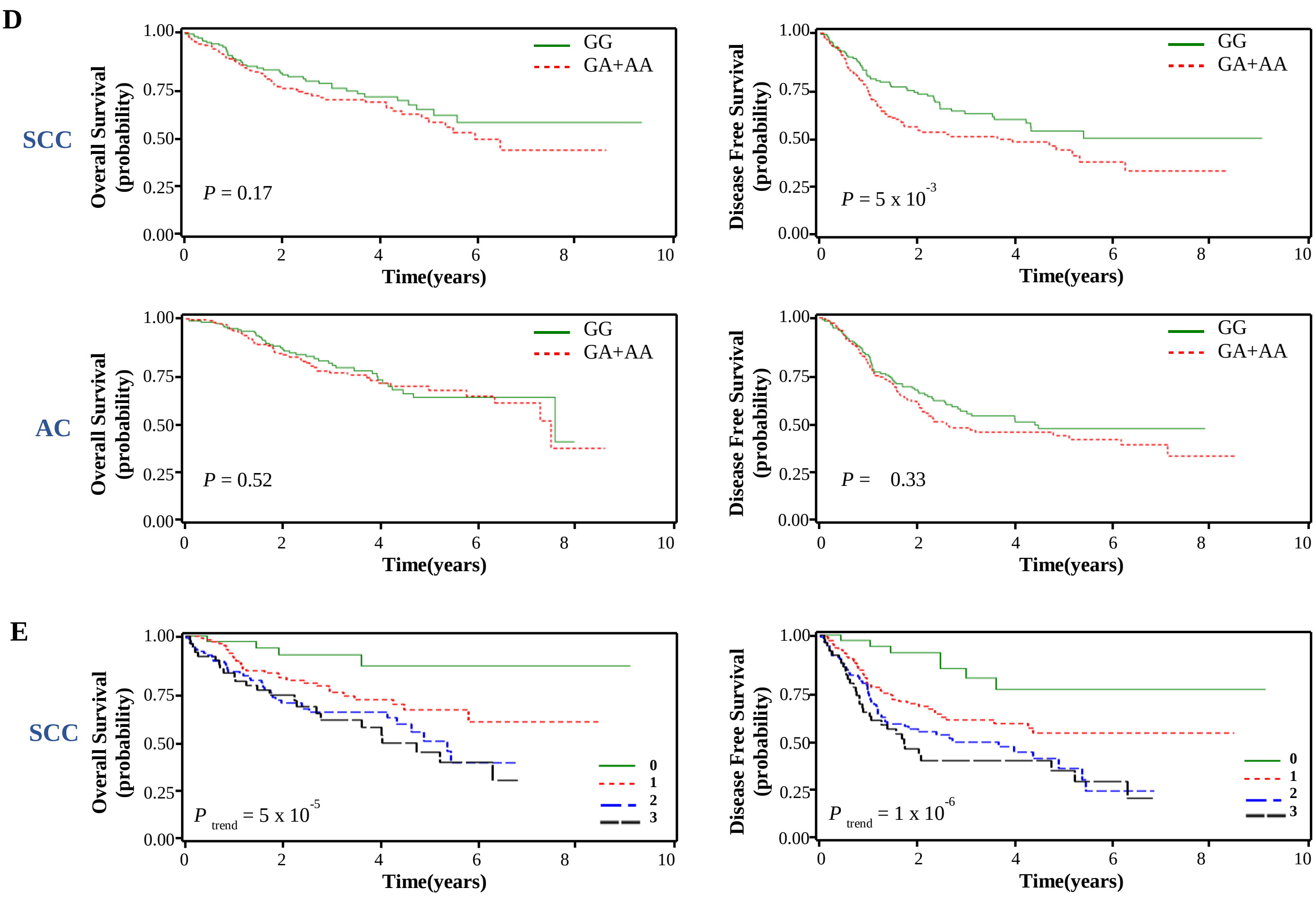

Supplementary Figure 1. Overall survival and disease free survival curves according to *ENO1* rs2274971A>G (A), *PFKM* rs11168417C>T (B), *PFKP* rs1132173C>T (C), and *PDK2* rs3785921G>A (D) genotypes in SCC and AC, and according to the number of bad genotypes of *ENO1* rs2274971A>G, *PFKM* rs11168417C>T, and *PDK2* rs3785921G>A (E). *P* values in the multivariate Cox hazard models. SCC, squamous cell carcinoma; AC, adenocarcinoma.
